# Neighborhood Racial Composition and Gun Homicides

**DOI:** 10.1001/jamanetworkopen.2020.27591

**Published:** 2020-11-30

**Authors:** Chaeyoung Cheon, Yuzhou Lin, David J. Harding, Wei Wang, Dylan S. Small

**Affiliations:** 1 Departmentof Statistics, University of Pennsylvania, Philadelphia; 2 Departmentof Sociology, University of California, Berkeley, Berkeley

## Abstract

This cross-sectional study examines disparities in gun homicide rates among neighborhoods of different racial composition for fixed levels of socioeconomic status.

## Introduction

Substantial racial disparities exist for gun homicide deaths in the US: the 2003 to 2017 age-adjusted rate was 7.4 times higher for Black individuals than for White individuals.^1^ Walker et al^2^ found an even greater disparity in Chicago and suggested that because race may be a surrogate for income, public health interventions should aim to alleviate poverty in order to reduce gun violence. However, in Philadelphia, Black individuals of the same income level as White individuals were at higher risk of being shot, suggesting that public policies to reduce gun violence and racial disparities might need to go beyond alleviating poverty.^3^ In this cross-sectional study, we examine disparities in gun homicide rates among neighborhoods of different racial composition for fixed levels of socioeconomic status.

## Methods

Institutional review board approval was not sought because this study used secondary public use data files. Primary patient data was not collected, so informed consent was not sought, in accordance with 45 CFR §46. This study follows the Strengthening the Reporting of Observational Studies in Epidemiology (STROBE) reporting guideline for observational studies.

We obtained from the Gun Violence Archive information on gun homicide deaths by US Census tract from 2014 to 2018. To measure US Census tract racial composition and socioeconomic status, we used data from the American Community Survey’s 2014 to 2018 US Census tract profiles and computed the deprivation index, which is a 0 to 1 scale based on poverty rate; median household income; residents older than 24 years without a high school degree; residents without health insurance; residents receiving public assistance income, food stamps, or the Supplemental Nutrition Assistance Program; and vacant housing units.^4^ We removed US Census tracts with missing data (1.4%).

We estimated a generalized additive model with a quasi-Poisson distribution using the mgcv package in R statistical software version 3.6.3 (R Project for Statistical Computing). The response was US Census tract 2014 to 2018 gun homicide deaths, and the explanatory variables were proportion of Black residents and deprivation index. The model considered interactions between the 2 explanatory variables and smoothed the effects of the variables. An offset of log population size accounted for variation in US Census tracts’ population sizes. Data analysis was performed from January to September 2020.

## Results

There were 71 499 people killed by guns in 2014 to 2018 across 72 041 US Census tracts.^1^ Panel A of the Figure shows the association between the proportion of Black residents and the deprivation index in US Census tracts. The median deprivation index tends to increase as the proportion of Black residents increases, but there is considerable overlap in the distributions and there are high and low deprivation index neighborhoods for all levels of proportion of Black residents. Panel B of the Figure shows the estimated mean incidence rate of gun homicides deaths per 1000 people per year over 2014 to 2018 by a US Census tract’s proportion of Black residents and deprivation index. The Table shows estimates and 95% CIs for several deprivation index and proportion of Black residents levels. For both high and low deprivation index levels, gun homicide deaths increased with the proportion of Black residents. For example, for moderately well-off neighborhoods (33rd percentile of deprivation index), the mean incidence rate per 1000 people per year increased from 0.017 (95% CI 0.016-0.018) in a 1% Black neighborhood to 0.077 (95% CI, 0.069-0.086) in a 90% Black neighborhood.

**Figure.**
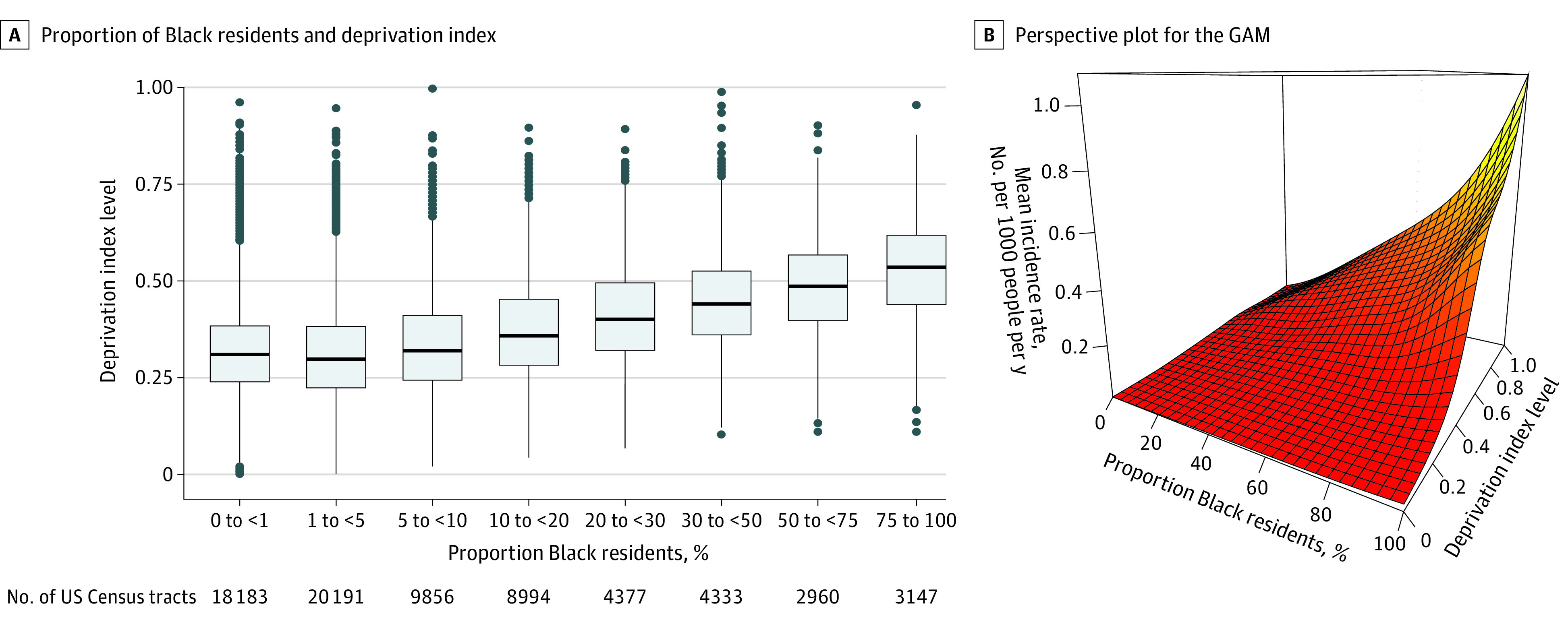
Deprivation Index and Estimated Mean Incidence Rate of Gun Homicide Deaths Panel A shows box plots of deprivation index in US Census tracts with different ranges of proportion Black residents. Horizontal lines within boxes denote medians, tops and bottoms of boxes denote 75th and 25th percentiles, error bars denote 1.5 interquartile ranges beyond the 75th and 25th percentiles, and dots denote outliers. Panel B shows a perspective plot for mean incidence rates per 1000 people per year from 2014 to 2018 (*z*-axis) by a US Census tract deprivation index level (*y*-axis) and proportion Black residents (*x*-axis) from the generalized additive model (GAM).

**Table.** Mean Incidence Rate of Gun Homicide Deaths per 1000 People per Year Over 5-Year Period From 2014 to 2018 by a US Census Tract’s Deprivation Index Level and Percentage Black Residents

Percentile, deprivation index level^a^	Point estimate (95% CI)			
	1% Black residents	13% Black residents	50% Black residents	90% Black residents
10th, 0.20	0.010 (0.009-0.010)	0.016 (0.014-0.017)	0.029 (0.025-0.033)	0.048 (0.041-0.056)
33rd, 0.28	0.017 (0.016-0.018)	0.025 (0.023-0.026)	0.047 (0.043-0.051)	0.077 (0.069-0.086)
50th, 0.34	0.022 (0.021-0.023)	0.033 (0.031-0.034)	0.063 (0.059-0.068)	0.109 (0.100-0.117)
67th, 0.40	0.029 (0.028-0.030)	0.041 (0.039-0.043)	0.087 (0.082-0.092)	0.168 (0.159-0.177)
99th, 0.72	0.059 (0.054-0.064)	0.118 (0.110-0.126)	0.255 (0.237-0.272)	0.511 (0.486-0.536)

^a^ A higher deprivation index means more deprived, so the 10th percentile of deprivation index means a relatively well-off neighborhood, whereas the 90th percentile means an impoverished neighborhood.

## Discussion

For a fixed socioeconomic status of a US Census tract—high, medium or low—US Census tracts with a higher proportion of Black residents have higher gun homicide rates. The US remains highly residentially segregated by race despite improvements since the 1960s.^5^ Besides residential segregation reducing Black individuals’ socioeconomic status by such mechanisms as inhibiting wealth accumulation through housing value and limiting access to high-quality schools,^6^ our findings suggest that even among neighborhoods of the same socioeconomic status, residential segregation may put Black individuals at higher risk of gun homicide. Potential explanations include the following being more prevalent in higher proportion Black neighborhoods: lack of institutional resources and opportunities caused by racial wealth gaps and underinvestment, the legacy of punitive law enforcement leading to difficulties controlling crime, lower collective efficacy due to lack of political power or city responsiveness, geographic proximity to poor neighborhoods, and gang networks or interconnections. Further studies should be conducted to investigate these explanations and design policies to reduce gun homicides. Limitations of this study include the inability to pinpoint the demographic characteristics of the people affected by gun homicides.
